# Supplementary material for: Uncovering the molecular mechanism for dual effect of ATP on phase separation in FUS solution
Source: Sci Adv. 2022 Sep 14;8(37):eabo7885. doi: 10.1126/sciadv.abo7885 (PMC9473584; doi:10.1126/sciadv.abo7885)
Supplement: Supplementary file 1 — Supplementary Text Figs. S1 to S4 [file sciadv.abo7885_sm.pdf]

Science Advances
AAAS

Supplementary Materials for

Uncovering the molecular mechanism for dual effect of ATP on phase separation in FUS solution

Chun-Lai Ren *et al.*

Corresponding author: Chun-Lai Ren, chunlair@nju.edu.cn; Hong-Ming Ding, dinghm@suda.edu.cn; Yu-Qiang Ma, myqiang@nju.edu.cn

Sci. Adv. **8**, eabo7885 (2022)
DOI: 10.1126/sciadv.abo7885

The PDF file includes:

Supplementary Text
Legends for movies S1 to S3
Figs. S1 to S4

Other Supplementary Material for this manuscript includes the following:

Movies S1 to S3

I: SUPPLEMENTARY TEXT

The mean-field theory for FUS solutions without ATP

In the case of FUS proteins, the total free energy can be written as:

$$\beta F_{tot} = \frac{\phi}{N_1}\ln\phi + \frac{(1-\phi)}{N_2}\ln(1-\phi) + \frac{m_1\phi}{N_1}[p\ln p + (1-p)\ln(1-p)] + \frac{m_2\phi}{N_1}(1-p\frac{m_1}{m_2})\ln(1-p\frac{m_1}{m_2}) - p\frac{m_1\phi}{N_1}(\ln\frac{m_2\phi}{N_1 e} + \beta\epsilon_1) \tag{S1}$$

The equilibrium fraction of Tyr-Arg binding can be obtain from $\partial\beta F_{tot}/\partial p = 0$, which gives

$$\ln p - \ln(1-p) - \ln(1-p\frac{m_1}{m_2}) - \ln\frac{m_2\phi}{N_1} - \beta\epsilon_1 = 0 \tag{S2}$$

The minimized thermodynamic potential is given by:

$$\beta F_{min} = \frac{\phi}{N_1}\ln\phi + \frac{(1-\phi)}{N_2}\ln(1-\phi) + \frac{m_1\phi}{N_1}\ln(1-p) + \frac{m_2\phi}{N_1}\ln(1-p\frac{m_1}{m_2}) + p\frac{m_1\phi}{N_1} \tag{S3}$$

To study the phase behavior, we calculate chemical potential ($\mu = \frac{\partial\beta F_{min}}{\partial\phi}$) and osmotic pressure ($\Pi = \phi\mu - \beta F_{min}$), which are written as:

$$\mu = \frac{1}{N_1}\ln\phi + \frac{1}{N_1} - \frac{1}{N_2}\ln(1-\phi) - \frac{1}{N_2} + \frac{m_1}{N_1}\ln(1-p) + \frac{m_2}{N_1}\ln(1-p\frac{m_1}{m_2}) \tag{S4}$$

$$\Pi = \frac{\phi}{N_1} - \frac{\phi}{N_2} - \frac{1}{N_2}\ln(1-\phi) - pm_1\frac{\phi}{N_1} \tag{S5}$$

The binodal curve can be obtained form $\mu_1 = \mu_2$ and $\Pi_1 = \Pi_2$, where subscripts 1 and 2 refer to the two coexisting phases: a dilute phase and a condensed phase. In the calculation, the protein volume fraction changes from close to 0 to close to 1. And we calculate the chemical potential and osmotic pressure for each system with a certain protein concentration. Then we can obtain the curve as functions of the chemical potential and osmotic pressure. The coexisting phases correspond to the intersection of the curve. The phase diagram is composed of different coexisting phases.

The mean-field theory for FUS solutions with ATP

In the case of FUS proteins with the introduction of ATP, the total free energy can be written as:

$$\begin{aligned}\beta\mathscr{F}_{tot} = &\frac{\phi}{N_1}\ln\phi + \frac{(1-\lambda\phi-\phi)}{N_2}\ln(1-\lambda\phi-\phi) + \lambda\phi\ln\lambda\phi + \chi\lambda\phi(1-\phi-\lambda\phi) - \lambda\phi\mu_{ATP} \\ &+ \frac{m_1\phi}{N_1}[p\ln p + (1-p)\ln(1-p)] + L\lambda\phi[q\ln q + (1-q)\ln(1-q)] \\ &+ \frac{m_2\phi}{N_1}(1-\frac{pm_1}{m_2}-\frac{qL\lambda N_1}{m_2})\ln(1-\frac{pm_1}{m_2}-\frac{qL\lambda N_1}{m_2}) \\ &-p\phi\frac{m_1}{N_1}(\ln\frac{m_2\phi}{N_1}-\beta\epsilon_1-1) - qL\lambda\phi(\ln\frac{m_2\phi}{N_1}-\beta\epsilon_2-1)\end{aligned} \tag{S6}$$

where μ_{ATP} is obtained from the reservoir. The reservoir is composed of solvent and ATP. The volume fraction of ATP in the reservoir is represented by φ. The free energy of the reservoir is given by

$$\beta F_{reser} = \frac{(1-\varphi)}{N_2}\ln(1-\varphi) + \varphi\ln\varphi + \chi\varphi(1-\varphi) - \varphi\mu_{ATP} \tag{S7}$$

After doing $\frac{\partial\beta F_{reser}}{\partial\varphi} = 0$, we can get $\mu_{ATP} = \ln\varphi - \frac{1}{N_2}\ln(1-\varphi) - \frac{1}{N_2} + 1 + \chi(1-2\varphi)$

Minimizing the total free energy, we obtain:

$$\ln p - \ln(1-p) - \ln(1-p\frac{m_1}{m_2} - q\frac{L\lambda N_1}{m_2}) - \ln\phi\frac{m_2}{N_1} + \beta\epsilon_1 = 0 \tag{S8}$$

$$\ln q-\ln(1-q)-\ln(1-p\frac{m_1}{m_2}-q\frac{L\lambda N_1}{m_2})-\ln\phi\frac{m_2}{N_1}+\beta\epsilon_2=0 \tag{S9}$$

$$\frac{1}{N_2}\ln(1-\lambda\phi-\phi)+\frac{1}{N_2}-\ln\lambda\phi+\mu_{ATP}-1-\chi(1-2\lambda\phi-\phi)-L\ln(1-q)=0 \tag{S10}$$

Now the minimized thermodynamic potential becomes:

$$\begin{aligned}\beta\mathscr{F}_{min}=&\frac{\phi}{N_1}\ln\phi+\frac{(1-\phi)}{N_2}\ln(1-\lambda\phi-\phi)-\lambda\phi+\frac{\lambda\phi}{N_2}+\phi\frac{m_1}{N_1}\ln(1-p)\\&+\phi\frac{m_2}{N_1}\ln(1-p\frac{m_1}{m_2}-q\frac{L\lambda N_1}{m_2})+p\phi\frac{m_1}{N_1}+qL\lambda\phi+\chi(\lambda\phi)^2\end{aligned} \tag{S11}$$

To obtain phase diagram, we need to calculate the chemical potential and osmotic pressure. After doing the first derivative of the minimum free energy, we get:

$$\begin{aligned}\mu=&\frac{1}{N_1}\ln\phi+\frac{1}{N_1}-\frac{1}{N_2}\ln(1-\lambda\phi-\phi)-\frac{1-\phi}{N_2(1-\lambda\phi-\phi)}+\frac{m_1}{N_1}\ln(1-p)\\&+\frac{m_2}{N_1}\ln(1-\frac{pm_1}{m_2}-\frac{qL\lambda N_1}{m_2})+\frac{\lambda Lq}{(1-\frac{pm_1}{m_2}-\frac{qL\lambda N_1}{m_2})}+\frac{pm_1}{N_1}\\&-(\frac{\partial\lambda}{\partial\phi}\phi+\lambda)\left[\frac{\lambda\phi}{N_2(1-\lambda\phi-\phi)}+1+\frac{Lq(\frac{pm_1}{m_2}+\frac{qL\lambda N_1}{m_2})}{(1-\frac{pm_1}{m_2}-\frac{qL\lambda N_1}{m_2})}-2\chi(\lambda\phi)\right]\\&-\frac{\partial p}{\partial\phi}\left[\frac{\phi m_1}{N_1(1-\frac{pm_1}{m_2}-\frac{qL\lambda N1}{m_2})}+\frac{\phi m_1 p}{N_1(1-p)}\right]-\frac{\partial q}{\partial\phi}\left[\frac{L\phi\lambda(\frac{pm_1}{m_2}+\frac{qL\lambda N_1}{m_2})}{(1-\frac{pm_1}{m_2}-\frac{qL\lambda N_1}{m_2})}\right]\end{aligned} \tag{S12}$$

where $\frac{\partial p}{\partial\phi}$, $\frac{\partial q}{\partial\phi}$ and $\frac{\partial\lambda}{\partial\phi}$ can be obtained from Eqs. (S8)-(S10). Once we have the chemical potential, the osmotic pressure can be obtained from $\Pi=\phi\mu-\beta\mathscr{F}_{min}$. The criteria of $\mu_1=\mu_2$ and $\Pi_1=\Pi_2$ ensures the binodal curve of the phase diagram.

II: MOVIES S1 TO S3

Movie S1. The exchange of protein chains between the condensate and the surrounding in the absence of ATP.

Movie S2. The exchange of protein chains between the condensate and the surrounding in the presence of ATP (n_{ATP}=30).

Movie S3. The exchange of protein chains between the condensate and the surrounding in the presence of ATP (n_{ATP}=70).

III: FIGS S1 TO S4

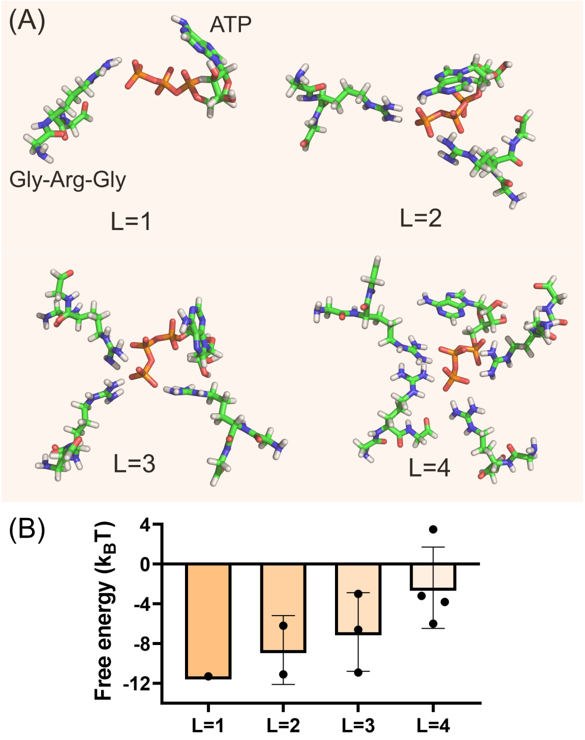

Fig. S1. The SQM result for the ATP-tripeptide (i.e, Gly-Arg-Gly) binding. (A) Snapshots for the typical binding modes between one ATP and one/several tripeptide(s), where the tripeptide(s) all bind to the triphosphate part of the ATP. (B) The binding free energy of the ATP-tripeptide interaction in the above cases.

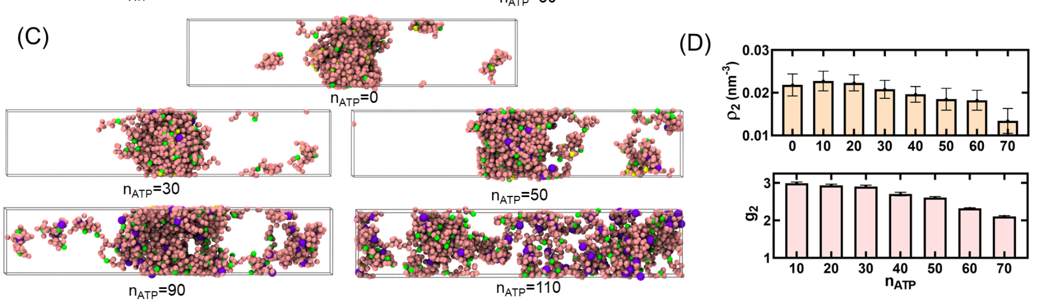

Fig. S2. The morphology change induced by the addition of ATP in CGMD simulation. (A) Final snapshots for the FUS solution under the dilute condition ($n_{pro}=20$) in the CGMD simulations. (i) and (ii) are two cases of L$>$1 and L=1. The proteins are described as pink chains, the yellow and green beads represent Arg and Tyr residues respectively, the purple beads stands for the ATP molecules. n_{ATP} is the number of ATP molecules in simulations. (B) The corresponding protein density concentration distributions along the x direction in (A). (C) Final snapshots for the FUS solution under the dense condition ($n_{pro}=50$) in the CGMD simulations. Here, we only consider the case of L$>$1. (D) The concentration of protein droplets and the number of Arg residues per ATP as functions of the ATP number under the dense condition.

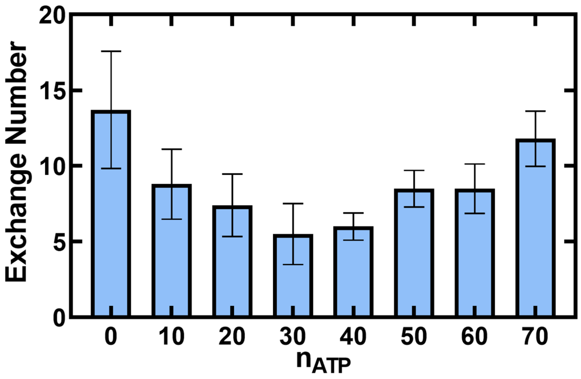

Fig. S3. The average number of proteins participating in the molecular exchange as a function of ATP number from CGMD simulations.

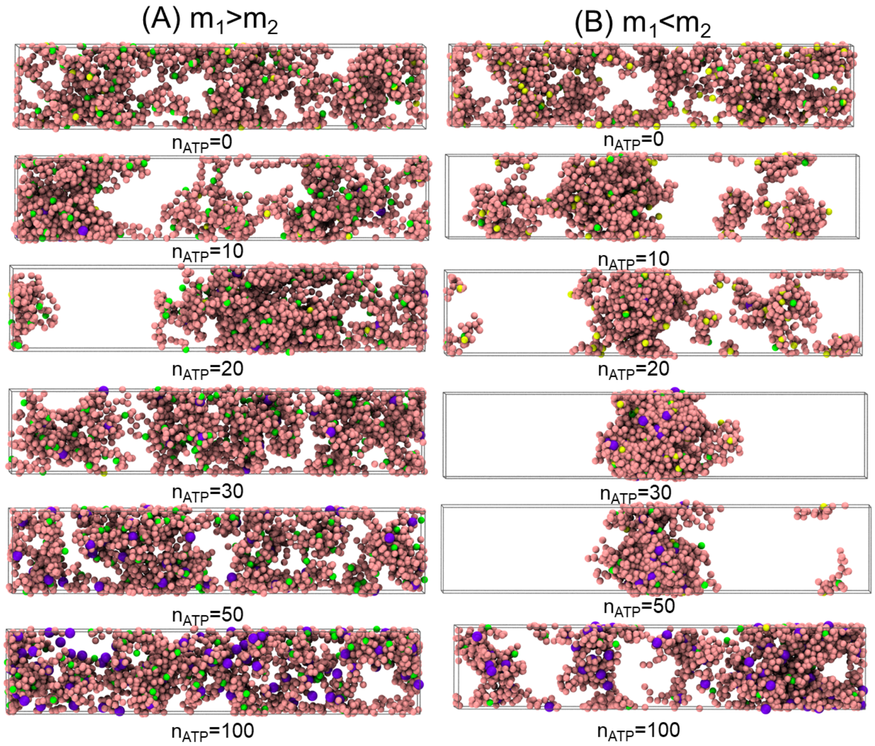

Fig. S4. Final snapshots for the protein solutions with asymmetric numbers of Tyr and Arg residues with the addition of different numbers of ATP in CGMD simulations. (A) $m_1 > m_2$, (B) $m_1 < m_2$. The number of protein chains in the simulations is 50. The proteins are described as pink chains, the yellow and green beads represent Arg and Tyr residues respectively, the purple beads stands for the ATP molecules.
